# Pyrazoles as Key Scaffolds for the Development of Fluorine-18-Labeled Radiotracers for Positron Emission Tomography (PET)

**DOI:** 10.3390/molecules25071722

**Published:** 2020-04-09

**Authors:** Pedro M. O. Gomes, Artur M. S. Silva, Vera L. M. Silva

**Affiliations:** LAQV-REQUIMTE, Department of Chemistry, University of Aveiro, 3810-193 Aveiro, Portugal; pm.gomes@ua.pt (P.M.O.G.); artur.silva@ua.pt (A.M.S.S.)

**Keywords:** positron emission tomography (PET), pyrazoles, fluorine-18, radionuclides, PET probes, imaging pharmaceuticals

## Abstract

The need for increasingly personalized medicine solutions (precision medicine) and quality medical treatments, has led to a growing demand and research for image-guided therapeutic solutions. Positron emission tomography (PET) is a powerful imaging technique that can be established using complementary imaging systems and selective imaging agents—chemical probes or radiotracers—which are drugs labeled with a radionuclide, also called radiopharmaceuticals. PET has two complementary purposes: selective imaging for diagnosis and monitoring of disease progression and response to treatment. The development of selective imaging agents is a growing research area, with a high number of diverse drugs, labeled with different radionuclides, being reported nowadays. This review article is focused on the use of pyrazoles as suitable scaffolds for the development of ^18^F-labeled radiotracers for PET imaging. A brief introduction to PET and pyrazoles, as key scaffolds in medicinal chemistry, is presented, followed by a description of the most important [^18^F]pyrazole-derived radiotracers (PET tracers) that have been developed in the last 20 years for selective PET imaging, grouped according to their specific targets.

## 1. Introduction

Positron emission tomography (PET) is a nuclear medicine functional imaging technique that uses gamma rays (formed as a result of the annihilation of the emitted positrons) to provide three-dimensional images that give information about the functioning of a person’s specific organs. PET is based on the detection of tiny amounts (picomolar) of a biological substance labeled with a short-lived positron-emitting radionuclide (PET tracer) without disturbing the biological system and has the advantage of being a noninvasive, functional and highly sensitive technique which provides a great wealth of information [1]. The PET tracers are drugs or biomolecules labeled with radionuclides such as ^11^C, ^13^N, ^15^O, ^18^F, ^64^Cu, ^68^Ga, ^89^Zr and ^124^I. Since radionuclides can replace the stable analogues, the PET probes have the same chemical structure as the drugs and biomolecules without altering their biological activity. The choice of the PET radionuclide must follow some criteria: (i) physical and chemical characteristics, (ii) availability and (iii) timescale of the biological process in the study. For different biological processes, some radionuclides are better than others. If the biological process in the study gives results hours or days after the injection of PET probe, ^64^Cu, ^89^Zr or ^124^I can be used, because these three radionuclides have a long half-life time (12.8 h, 78.4 h and 4.17 days, respectively). On the contrary, radionuclides as ^11^C or ^18^F are used for labeling small organic compounds for faster biological processes [1]. To avoid unnecessary radiation, it is important to choose a radionuclide with a half-life that enable the reaction of the radionuclide with the carrier molecule and matches the timescale of the biological process in the study. ^18^F is the ideal radionuclide for routine PET imaging, because it has a short half-life (109.8 min) but enough to allow all the process of synthesis, transport and imaging. Furthermore, it is a 97% positron-emitter, and the low positron energy of ^18^F leads to a high resolution of PET imaging [1,2].

In the last three decades, the interest in PET has been growing, and nowadays, this technique is used in several areas of medicine, such as oncology [3], cardiology [4], neurology [5] and also in drug development and evaluation [6]. Besides the aforementioned advantages of PET, there are also some limitations; pregnant women should not undergo in PET imaging; the radioactivity of PET tracers limits the number of times one patient can undergo PET scans and is a very expensive treatment [7]. Moreover, the potential of PET strongly depends on the availability of suitable PET radiotracers. For instance, 2-[^18^F]fluoro-2-deoxyglucose ([^18^F]FDG) is one of the most used radiotracers for the clinical diagnosis and evaluation of cancer. However, it has inherent drawbacks, namely its high accumulation in inflamed and infected tissues giving false-positive results and its low uptake in tumors that grow slowly, which can lead to false-negative results [8]. Finally, the development of new PET-imaging probes is not trivial, and radiochemistry is a major limiting factor for this field. Hence, the research and development of new PET tracers is a challenging issue of major importance within the scientific community.

Following our interest in the synthesis and biological evaluation of pyrazole-type compounds envisaging their use in medicine [9,10,11,12,13,14] and considering their huge potential for application in PET field, herein we present an overview of pyrazole-derived PET radiotracers developed in the last 20 years. Pyrazoles labeled with ^18^F radionuclide will be the focus of this review article, given the aforementioned advantages of this radionuclide for routine PET imaging.

## 2. Pyrazoles

**Figure 1:**
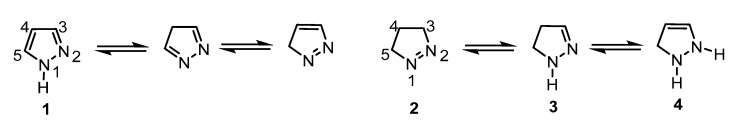
Chemical structures and numbering of pyrazole (**1**) and dihydropyrazole (pyrazoline) tautomers **2**–**4** [18].

This family of compounds is characterized by a simple heteroaromatic five-membered ring containing three carbon and two nitrogen atoms in adjacent positions [15]. The occurrence of the pyrazole core in nature is rare, probably due to the difficulty of living organisms to make a N-N bound [16]. Until now, there are only approximately 20 natural compounds with a pyrazole core on their structures [17]. Although scarce in nature, pyrazole (1*H*-pyrazole (**1**), Figure 1) and its reduced derivatives (pyrazolines **2**–**4**, Figure 1) are considered privileged structures in medicinal chemistry. Pyrazole scaffold is present in several synthetic drugs, being celecoxib (Celebrex^®^), sildenafil (Viagra^®^), rimonabant, fomepizole, penthiopyrad and sulfaphenazole the most notorious [15].

Pyrazole derivatives act on diverse and relevant biological targets, which make them attractive for the development of PET tracers, and possess a wide range of pharmaceutical activities. Abdel-Maksoud et al. have demonstrated antitumoral activity of several compounds with a pyrazole and sulfonamide moieties [19]; antibacterial and antifungal activity of pyrazole was demonstrated by Chowdary et al. [20]. Pyrazoles also showed good activity as monoamine oxidase inhibitors, antidepressant and anticonvulsant agents [21], as BRAF inhibitors [22] and DNA gyrase inhibitors [23] and present antileishmanial, anti-inflammatory, analgesic, antidiabetic and cannabinoid activity; cyclin-dependent kinase and tissue-nonspecific alkaline phosphatase inhibitory activity and moderate antihepatotoxic activity, among others [9,11,24,25,26,27,28,29]. In 2013, Prabhu et. al. reported the antioxidant activity of pyrazole [30]. They demonstrated that pyrazole (1,2-diazole) can be used to prevent nephrotoxicity caused by cisplatin, a drug used to treat several cancers. Cisplatin provokes renal damage because of its toxicity to proximal tubule cells and can reduce glomerular filtration, resulting in renal failure. One of the reasons of nephrotoxicity induced by cisplatin is the decreasing concentration of glutathione (GSH). Pyrazole prevents nephrotoxicity induced by cisplatin by counteracting this effect, increasing the concentration of this enzyme [30]. Recently, Silva et al. reported the antioxidant activity of around three hundred pyrazoles [18].

### 2.1. Pyrazoles as Probes for PET Imaging

**Figure 2:**
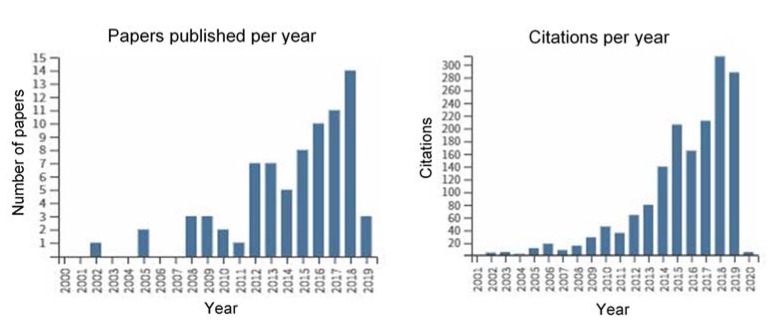
Number of papers published and citations per year found using the keywords “pyrazoles” and “positron emission tomography” and “^18^F” in Web of Science in the period 2000–2020.

The labeling of pyrazole-type compounds with ^18^F for PET imaging is a research issue that has been growing significantly, as evidenced by the number of papers and citations in the last years, according to our search on Web of Science using the keywords (pyrazole) and (positron emission tomography) and (^18^F) (Figure 2). In this section are described the most important [^18^F]-labeled pyrazoles developed for use as PET radiotracers. The compounds are presented according to their specific targets, which are indicated by alphabetical order and not by their relevance in the PET field. Whenever possible, the most promising applications of these compounds in this field are discussed.

#### 2.1.1. Adenosine Receptors Ligands

**Figure 3:**
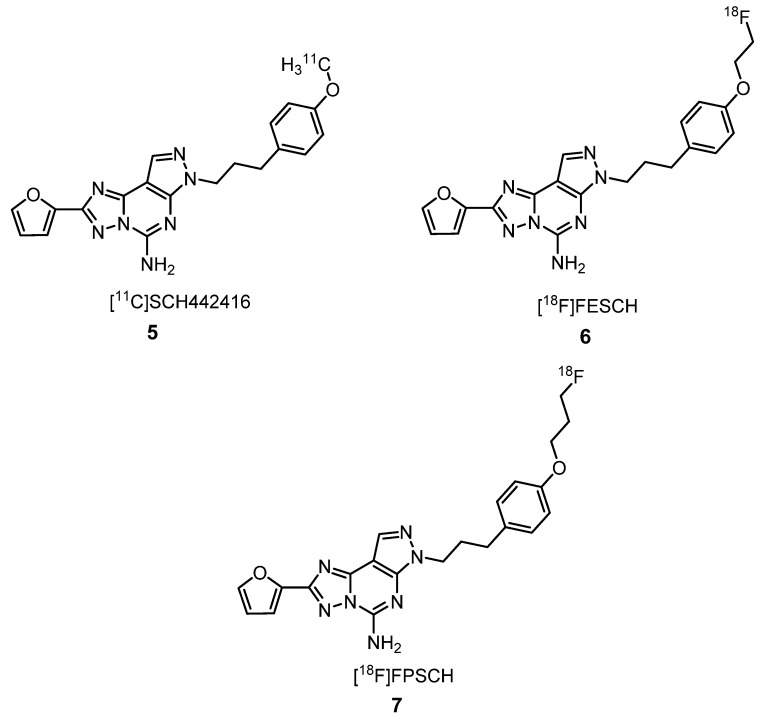
Adenosine receptor (A_2A_R) radioantagonists **5**–**7** for PET imaging [32,33].

Adenosine, an endogenous-signaling substance, is a purine ribonucleoside composed of adenine (purine base) and ribose (sugar molecule), which is produced in response to metabolic stress and cell damage. It induces several physiopathological effects, regulating the central nervous, cardiovascular, peripheral, and immune systems due to the rapid generation of adenosine from cellular metabolism and the widespread distribution of its receptor subtypes in almost all organs and tissues [31]. There are four types of adenosine receptors: A_1_, A_2A_, A_2B_ and A_3_. A_2A_R are abundant in dopamine-rich regions of the brain, being the striatum the region with a higher concentration of A_2A_R [31]. In preclinical studies, A_2A_R antagonists showed potential benefits in the treatment of some neurodegenerative diseases like Alzheimer’s disease (AD) and Parkinson’s disease (PD), neuroinflammation, ischemia, spinal cord injury, drug addiction and other conditions [31]. Khanapur et al. developed a pyrazolo[4,3-*e*]-1,2,4-triazolo[1,5-*c*]pyrimidine labeled with carbon-11, [^11^C]SCH442416 (**5**) and its [^18^F]fluoroethyl and [^18^F]fluoropropyl derivatives ([^18^F]FESCH (**6**) and [^18^F]FPSCH (**7**), respectively) (Figure 3), and both radioligands showed a distribution in the rat brain corresponding to the regional A_2A_R densities, as evidenced by in vitro autoradiography (ARG) experiments and binding assays [32]. These two tracers showed a similar ratio specific/nonspecific binding, using the striatum as the specific binding and cerebellum as the nonspecific binding (4.6 at 25 and 37 min after injection, respectively) and reversible binding in rat brains, although their kinetics were slightly different [32]. Recently, the same authors studied the full kinetics of radioligands **6** and **7** in rat brains. On the basis of the Akaike information criterion, they have found that 1TCM was the most appropriate model for describing [^18^F]FPSCH kinetics, whereas 2TCM was the most suitable model for [^18^F]FESCH kinetics. Using dynamic PET imaging, under baseline and full blocking conditions, they proved that **6** is the most suitable PET radioligand for quantifying A_2A_R receptor expression in the rat brain. However, before starting the clinical use of **6**, it will be necessary to reevaluate the brain uptake in humans due to possible interspecies differences in tracer kinetics and metabolisms [33].

#### 2.1.2. Cannabinoid Receptors Ligands

Products derived from *Cannabis sativa* are some of the oldest and widely used drugs in the world. These products are known as natural cannabinoids, but several synthetic cannabinoids have been developed as well. Cannabinoids have been used as analgesics for more than 100 years [34]. Additionally, they have been used as antiemetic agents to prevent chemotherapy-induced nausea and vomiting, because they can bind to opiate receptors in the forebrain, blocking the vomiting center in the medulla [35]. In 1998, Williams and Kirkham have demonstrated that anandamide, an endogenous cannabinoid, provokes hyperphagia in satiated rats [36]. Cannabinoid-type compounds bind to cannabinoid receptors, which can be divided in two groups—cannabinoid receptor type 1 (CB_1_), predominantly found in the brain, and the peripheral cannabinoid receptor type 2 (CB_2_), mainly expressed in immune tissues, and both are G-protein-coupled membrane receptors [37].

**Figure 4:**
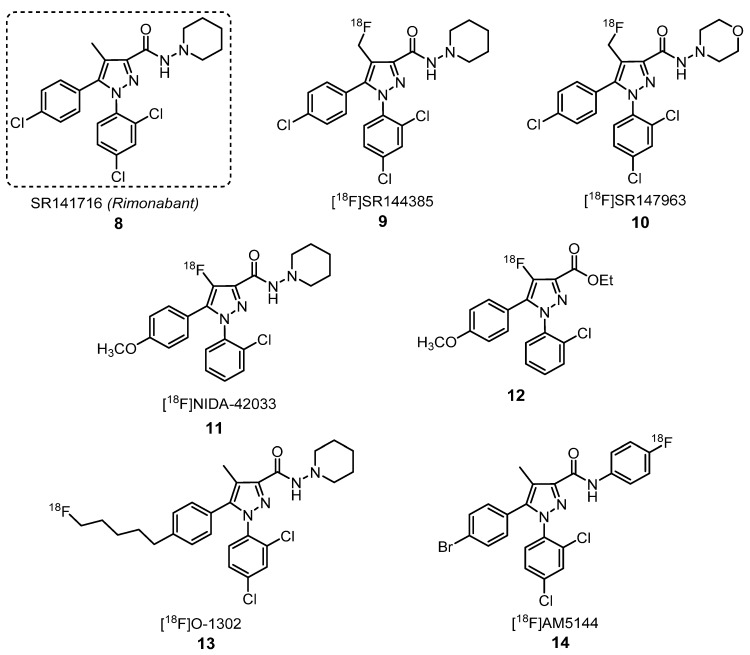
Structure of rimonabant (**8**) and structurally related [^18^F]pyrazole PET tracers **9**–**14** [37,39,41,42,43,44].

**Figure 5:**
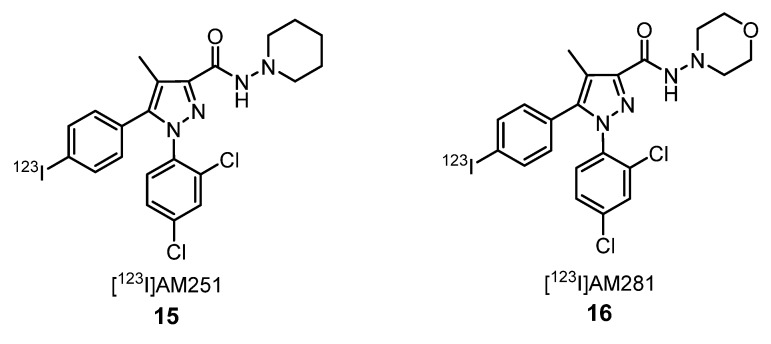
Pyrazole-derived PET tracers **15** and **16** labeled with ^123^I [44].

The imaging of the CB_1_ receptor is of great importance for studying its role in neuropsychiatric disorders, including depression, and in obesity, drug or alcohol addiction, and is an active target for in vivo imaging development [38]. The first selective CB_1_ receptor antagonist was [*N*-(piperidin-1-yl)-5-(4-chlorophenyl)-1-(2,4-dichlorophenyl)-4-methyl-1*H*-pyrazole-3-carboxamide] SR141716 (**8**), known as rimonabant (Figure 4) [39]. This compound was approved in Europe, in 2006, to treat obesity by reducing the patient’s appetite. Two years later, the European Medicines Agency (EMA) withdrew rimonabant from sale due to its evident secondary effects. Some studies demonstrated that SR141716 induced anxiety, depression, agitation, eating disorders, irritability, aggression and insomnia. Rimonabant was not approved in the USA by the Food and Drug Administration (FDA) [40]. However, some analogs of SR141716 (**8**) have been labeled with radionuclides for PET imaging. [^18^F]SR144385 (**9**) and [^18^F]SR147963 (**10**) [37,41], [^18^F]NIDA-42033 (**11**) and its related ester derivative (**12**) [42], [^18^F]O-1302 (**13**) [43] and [^18^F]AM5144 (**14**) [44] are some examples of PET tracers labeled with ^18^F (Figure 4), while [^123^I]AM251 (**15**) and [^123^I]AM281 (**16**) [44] are two examples of PET tracers labeled with ^123^I (Figure 5).

Studies were performed to evaluate the specificity of cannabinoids [^18^F]SR144385 (**9**) and [^18^F]SR147963 (**10**) (Figure 4) for CB_1_ receptors [37]. After 15 min of injection in male CD-1 mice (25–30 g), compound **10** showed a higher brain uptake compared to compound **9** (5.70% ID/g and 3.06% ID/g, respectively). The hippocampus was the brain region that exhibited the highest uptake of both tracers, followed by the striatum, cerebellum, frontal cortex, cortex, olfactory tubercles and hypothalamus. Both the brain stem and thalamus showed low uptakes of the tracers, and the thalamus showed the fastest decrease of %ID/g. These results are in accordance with the knowledge of CB_1_ receptors’ brain density in rats, which was found to be lower in the thalamus region. For evaluation of the target to nontarget ratio, the thalamus was used as indicator of nonspecific binding. Compounds **9** and **10** showed differences in the post-injection time they reached the maximum target to nontarget ratio (90 min for compound **9** with a ratio of 2.50 and 60 min for compound **10** with a ratio of 1.69) [37]. In vivo selectivity and specificity studies of [^18^F]SR144385 (**9**) showed that significant blocking of this tracer uptake was achieved when a 1-mg/kg dose of the structurally similar blocking agent SR141716 (**8**) was given 30 min prior to the radiotracer. Likewise, the uptake of [^18^F]SR147963 (**10**) at either 30 or 60 min post-injection was also blocked by a 1-mg/kg dose of SR141716 (**8**) given 30 min before the radiotracer.

In 2005, Li et al. synthetized the radioactive pyrazole [^18^F]AM5144 (**14**) (Figure 4) as a PET radioligand candidate for cannabinoid CB_1_ receptors. Using baboons, they demonstrated that the highest radioactivity concentration of compound **14** was found on the cerebellum, and the lowest concentration was found in the thalamus. They concluded that there was a poor brain uptake of **14** and of other related [^18^F]pyrazoles because of their high lipophilicity, although this property is not the key factor in brain uptake. This study proved that there is no close relationship between the clogP value and brain uptake, indicating that there are other uptake key factors [44]. Despite the high in vitro-binding affinity and moderate lipophilicity of [^18^F]O-1302 (**13**) (Figure 4), it is not suitable for imaging the CB_1_ receptor in the brain due to poor brain entry and high levels of nonspecific binding [43]. Although radiotracer uptake in the brain is often considered a function of the log P that peaks between a log P of 2 and 3, the investigation of the factors that affect brain uptake beyond lipophilicity is crucial to a better comprehension of this process. Some studies highlighted that there may be large species differences in brain penetration for a given PET radiotracer; for example, the brain uptake of [^123^I]AM251 (**15**) (Figure 5) is very different comparing mice and monkeys, being higher for mice [44,45].

**Figure 6:**
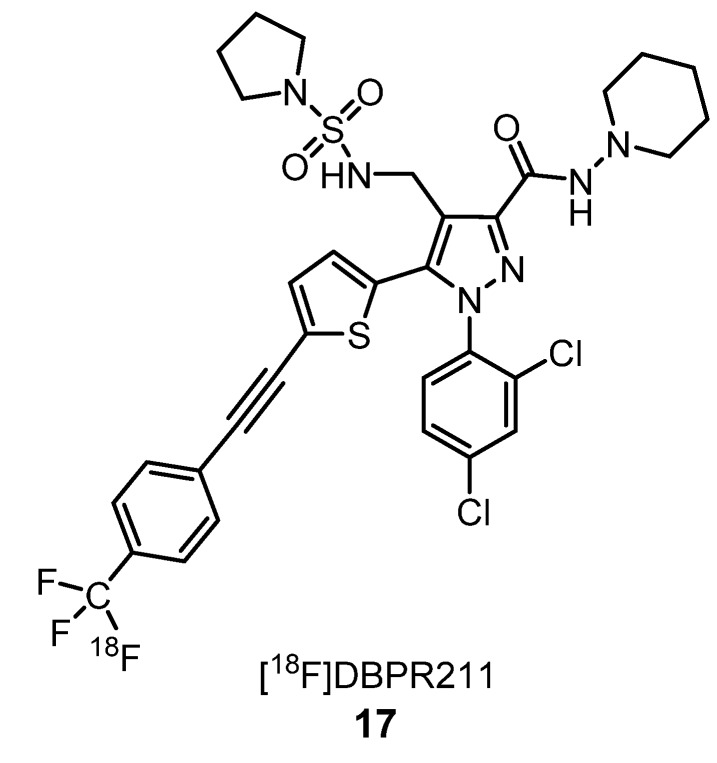
[^18^F]pyrazole-derived peripheral CB_1_ antagonist **17** [46].

Chang et al. observed that [^18^F]DBPR211 (**17**) (Figure 6) was distributed in the brain, liver, heart, thigh muscle and kidney when intravenously injected in mice. In the brain, it was found in a very low percentage (less than 1%) over 90-min scans. This result is evidence of its action as a peripherally restricted CB_1_ antagonist [46]. Taking into account these observations, this radiotracer can be seen as a promising tool to study metabolic processes associated with peripheral CB_1_ receptors.

#### 2.1.3. Cyclooxygenase Inhibitors

**Figure 7:**
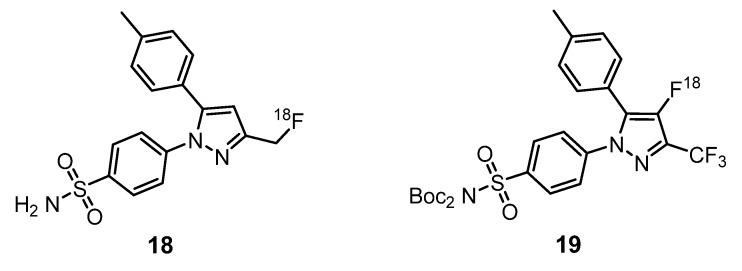
[^18^F]-labeled COX-2 inhibitors **18** and **19** [49,50].

Cyclooxygenase-2 (COX-2) is an enzyme with high-level expressions at sites of inflammation and cancer and is also a promising target for neuroinflammation imaging [47,48]. The development of agents capable of monitoring COX-2 levels is highly desirable for cancer prevention and therapy. To achieve this goal, Uddin et al. synthetized several indomethacin and celecoxib derivatives and evaluated their IC_50_ values for the inhibition of COX-2 in vivo and in intact cells [49]. Indomethacin derivatives were effective COX-2 inhibitors in intact cells (0.09–0.26 µmol/L), but the synthesis of the respective radiotracer has some drawbacks, because the *p*-chlorobenzoyl group is not stable in the conditions of radiochemical synthesis. Among celecoxib derivatives, the more effective COX-2 inhibitor was compound **18** with an IC_50_ value of 0.16 µmol/L. After the synthesis of ^18^F-**18** (Figure 7), in vivo biodistribution was studied in the inflamed and contralateral footpad of male Sprague Dawley rats. The major advantage of this inflammation model is the possibility to image the inflamed footpad in comparison with the noninflamed contralateral footpad. Compound **18** had a higher accumulation in the inflamed footpad in comparison with the noninflamed. The selective binding between **18** and COX-2 was proved with an assay using celecoxib to block COX-2 before the injection of **18.** To confirm the COX-2 specificity of **18**, COX-2-null mice were injected with carrageenan to promote inflammation. COX-2-null feet demonstrated a 1.08 ± 0.09 ratio of inflamed/noninflamed feet. On other hand, the wild-type mice group, used as the control, had an uptake ratio of 1.48 ± 0.04. The ability of **18** to target COX-2 in human tumor xenografts was also demonstrated using 1483 HNSCC cells and HCT116 cells injected in the left and right hip of female nude mice. In the COX-2-null HCT116 tumor cells, the uptake of **18** was minimal, while, in the 1483 HNSCC cells, a significative uptake of the radiotracer was observed. Once again, to prove that the difference in the uptake of **18** was related with the expression of COX-2, mice bearing 1483 xenografts were pretreated with celecoxib to block the active site of COX-2. There was a significative lower uptake of **18** for mice pretreated with celecoxib when compared with the control group [49].

Lebedev et al. developed a new, high-affinity ^18^F-COX-2 inhibitor **19** (Figure 7) that is radiolabeled directly on a heteroaromatic ring with the purpose to increase biodistribution and metabolic stability [50]. In vitro studies demonstrated a clear correlation between COX-2 expression and uptake of this radiotracer. Moreover, pharmacokinetic studies in healthy mice revealed no bone retention or defluorination within 2 h of injection, significant blood clearance, since the molecule is excreted from blood within an hour mainly through the hepatobiliary excretion pathway, crossing of blood-brain barrier (BBB) and no significant metabolites in major organs. Although these properties make this probe ideal for PET imaging, some aspects related to radiochemical synthesis severely limit the application of this compound as a probe. In fact, the use of Et_4_NF^+^4HF has limited the specific activity to 3 Ci/mmol, and the current decay-corrected radiochemical yield of 2%, although enough for preclinical studies and the production of a single patient dose, need further improvement to achieve the successful use of this compound as a PET tracer.

**Figure 8:**
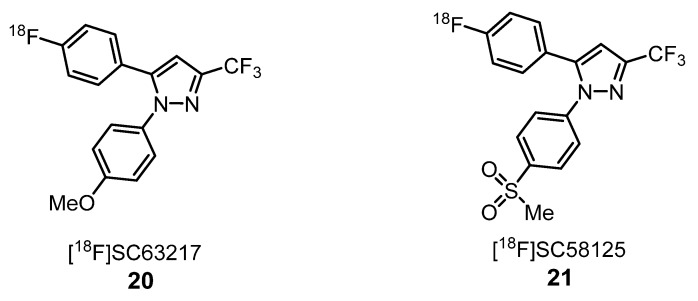
[^18^F]-labeled pyrazoles as probes for COX-1 **20** and COX-2 **21** [51].

Aiming to develop selective probes for the two COX enzyme subtypes, COX-1 and COX-2, McCharty et al. have prepared [^18^F]SC63217 (**20**) and [^18^F]SC58125 (**21**) (Figure 8), starting from the corresponding nonradioactive COX-1 and COX-2 selective inhibitors, SC63217 and SC58125, respectively [51]. Both compounds are structurally similar, presenting only a different substituent on only one aromatic ring. In vitro binding studies of both compounds, using J774 macrophages, revealed that compound **20** is a potential probe for COX-2, while **21** was not a good marker of COX-1 due to high nonspecific binding. In vivo studies showed that, for each tracer, rat biodistribution was well-matched with the known distribution of these enzymes [51].

#### 2.1.4. Dopamine Receptors Ligands

The subfamily of D_2_-like dopamine receptors includes the D_2_-, D_3_- and D_4_-receptor subtypes and mediates the action of dopamine in the brain by the inhibition of adenylate cyclase activity. While the distribution of D_2_- and D_3_- receptors is well-known, there are still some uncertainties regarding D_4_-receptor (D_4_R) expression. If D_4_R selective radioligands are available, PET can be suited to gain deeper knowledge into the distribution and pathophysiological role of D_4_R in humans.

**Figure 9:**
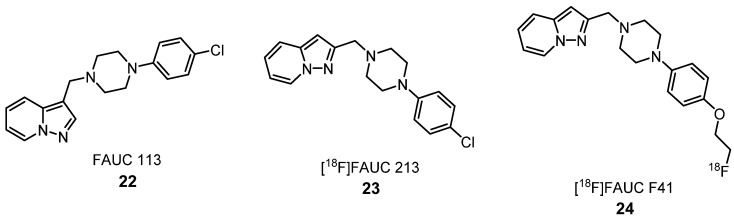
[^18^F]-labeled high-affinity dopamine receptor (D_4_R) tracers **22**–**24** [52].

In 2008, the pyrazolo[1,5-*a*]pyridine derivatives, FAUC 113 (**22**) and FAUC 213 (**23**) (Figure 9), bearing a (4-chlorophenyl)piperazinylmethyl moiety in the 3 and 2 positions of the pyrazolo[1,5-*a*]pyridine core, were synthetized. After an evaluation of their binding affinities for D_4_-like dopamine receptors, **22** and **23** were chosen as lead compounds for the development of [^18^F]-labeled PET tracers. A novel compound with a fluoroethoxyphenyl substituent in the *para*-position of the phenylpiperazinyl moiety showed the highest specificity to the D_4_ receptor. Due to these results, the next step was radiochemical synthesis to prepare the same labeled derivative, [^18^F]FAUC F41 (**24**) (Figure 9). Using coronal rat brains, in vivo AGR studies were performed to evaluate the specific binding of **24** to D_4_R. An increased uptake of this compound was detected in the hippocampus, hypothalamus, cortex, medial habenular nucleus and central medial thalamic nucleus. The observed binding pattern was mainly consistent with the known D_4_R distribution in the rat brains. The log P value for this compound was found to be 2.9, which may indicate adequate BBB penetration. Moreover, this ligand revealed high stability in human serum, even after long-term incubation for up to 90 min [52]. These results demonstrate that [^18^F]FAUC F41 (**24**) represents a potential radioligand for studying the D_4_R in vivo by PET imaging.

**Figure 10:**
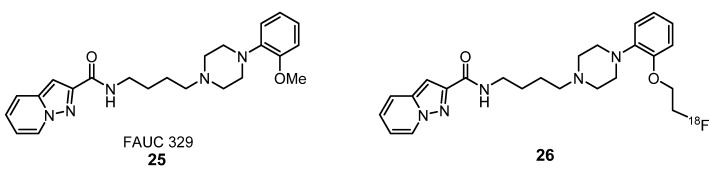
Radiotracer **26** derived from the dopamine D_3_ receptor ligand FAUC 329 (**25**) [53].

With the aim to develop a radiotracer with high selectivity and favorable lipophilicity for imaging of the D_3_-receptor in the brain, Stöβel et al. have synthetized compound **26** as the radioactive [^18^F]-labeled analogue of the D_3_ ligand FAUC 329 (**25**) (Figure 10) [53]. In vitro AGR studies showed that **26** successfully visualized D_3_-rich brain regions, including the islands of Calleja. However, in vivo PET imaging revealed that it does not significantly accumulate in the CNS structures of rat brains, probably due to a low BBB penetration. Instead, a significant uptake occurred in the brain ventricular system, due to a significant penetration of this compound in the blood-liquor barrier and, more noticeable, in the pituitary gland, outside the BBB [53]. The results of PET studies also suggest that the main mode of action of FAUC 329, in vivo, could be due to binding to the dopamine receptors in the pituitary gland.

#### 2.1.5. Glucocorticoid Receptor Ligands

**Figure 11:**
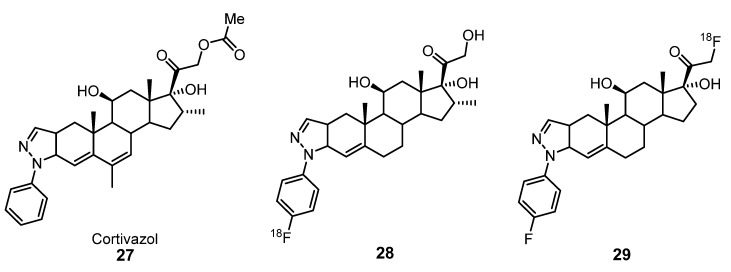
Cortivazol (**27**) and related [^18^F]arylpyrazolo steroids **28**–**29** as potential glucocorticoid receptor ligands for PET imaging [54,55].

In 1998, Hoyte et al. reported a series of aryl-pyrazolo steroids similar to the potent glucocorticoid cortivazol **27** and evaluated the affinity of these compounds for glucocorticoid receptors (GRs). Among them, the fluoro analog **28,** which showed good binding and was a very potent glucocorticoid, was labeled with ^18^F to be used as a glucocorticoid receptor-mediated imaging agent for PET (Figure 11) [54].

Later, Würst et al. used the 2′-(4-fluorophenyl)-21-[^18^F]fluoro-20-oxo-11β,17α-dihydroxypregn-4-eno[3,2-*c*]pyrazole (**29**) (Figure 11) as a ligand for studying brain GRs. Biodistribution and radiopharmacological studies with male Wistar rats revealed promising brain uptakes and low in vivo radiodefluorinations in comparison with other PET radioligands for brain GRs [55].

#### 2.1.6. Insulin-Like Growth Factor-1 Receptor Ligands

**Figure 12:**
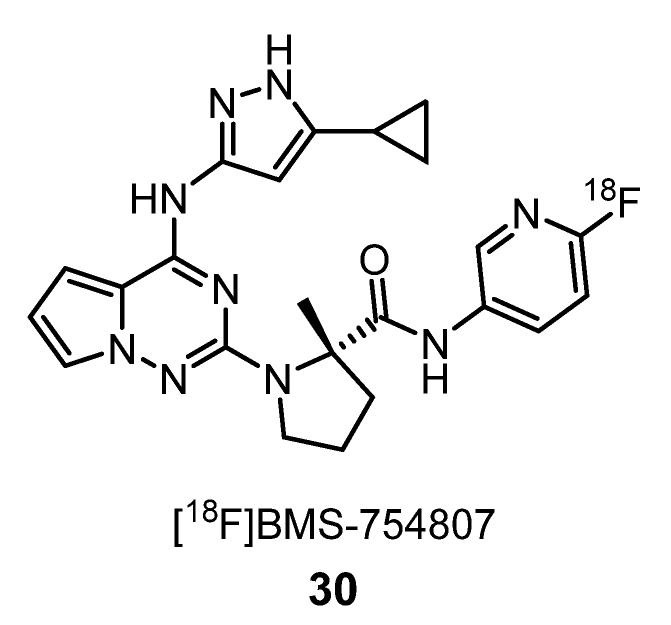
Potential PET radiotracer **30** for IGF-IR imaging [56].

Insulin-like growth factor-1 receptor (IGF-1R) is a potential therapeutic target, because it is overexpressed in many cancers, AD, traumatic brain injury, amyotrophic lateral sclerosis (ALS), Friedreich ataxia and aging. In 2013, Majo et al. synthetized and evaluated in vitro [^18^F]BMS-754807 (**30**) (Figure 12), a potent and reversible small molecule inhibitor of the IGF-1R/IR kinases’ family, currently in phase II clinical trials. ARG studies using surgically removed and pathologically identified grade IV glioblastoma, breast cancer and pancreatic tumors demonstrate that **30** binds to IGF-1R. The selectivity over other kinases, the presence of metabolically stable fluorine in the 2-substituted pyridine ring, which is amenable for radiolabeling by nucleophilic displacement with [^18^F]fluoride and a calculated lipophilicity (clogP) of 3.5, make this ligand a potential PET-imaging agent for in vivo monitoring of IGF-1R [56].

#### 2.1.7. Phosphodiester-10A Enzyme Inhibitors

Phosphodiester-10A (PDE10A) is an enzyme that hydrolyzes adenosine and/or guanosine 3′,5′-cyclic monophosphates (cAMP and cGMP, respectively). In the medium spiny neurons of the striatum, PDE10A messenger RNA and the corresponding protein are highly abundant. PDE10A inhibitors are a potential target for the diagnosis of schizophrenia, Huntington’s disease, PD, obsessive-compulsive disorder and addiction.

**Figure 13:**
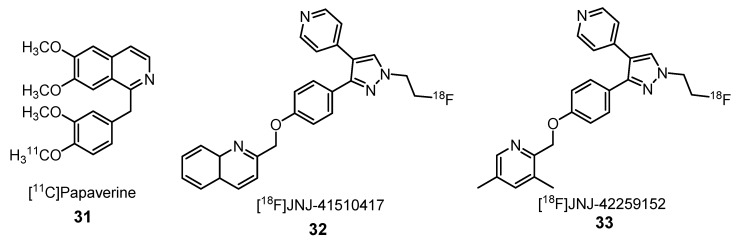
Radiotracers **31**–**33** for phosphodiester-10A (PDE10A) visualization in the brain by PET imaging [57,58,59,60,61,62].

In 2010, Tu et al. made a first attempt to visualize PDE10A using a ^11^C-radiolabeled PDE10A inhibitor named papaverine (**31**) (Figure 13). In vitro ^11^C-papaverine showed selective PDE10A binding, but in vivo failed because of rapid washout of the tracer [57]. To overcome this problem, Celen et al. synthetized a specific and selective radioligand for PDE10A, the ^18^F-quinoline-labeled [^18^F]JNJ-41510417 (**32**) (Figure 13). They used rats and PDE10A knockout mice to show that **32** binds specifically and reversibly to PDE10A in the striatum, presenting high accumulation therein. This brain region was the only to show an increase of tracer concentration after the injection (1.6 SUV after 2 min vs. 2.6 SUV after 60 min). Other brain regions, the hippocampus, cortex and cerebellum showed a washout of the radiotracer. These results are in accordance with the distribution of PDE10A. Despite the [^18^F]JNJ-41510417 (**32**) good target specificity and signal-to-noise characteristics, slow brain kinetics due to its high potency is a limitation [58].

Then, Laere et al., in collaboration with Janssen Pharmaceuticals, synthetized and studied the human biodistribution of another radioligand [^18^F]JNJ-42259152 (**33**) [2-(4-(1-(2[^18^F]fluoroethyl)-4-(4-pyridinyl)-1*H*-pyrazol-3-yl)phenoxy)methyl)-3,5-dimethylpyridine] (Figure 13), which belongs to a series of structurally related analogues of MP10 in which the 2-quinolinyl heterocycle was replaced with substituted 2-pyridinyl moieties, resulting in a slightly lower potency compared to [^18^F]JNJ-41510417 (**32**) [59,60]. Six healthy male Caucasians (23–69 years; 3 younger than 40 years and 3 older than 60 years) have been volunteers in the study managed by Laere. Compound **33** showed a rapid uptake in the striatum, a few minutes after the injection, with a high clearance rate consistent with specific binding in this target region. The results of this pilot study are in accordance with the distribution of PDE10A in the human brain and show a promising kinetics and biodistribution of [^18^F]JNJ-42259152 (**33**) [60].

Laere et al. have demonstrated that PDE10A activity in the brain can be reliably quantified using [^18^F]JNJ-42259152 (**33**) [61]. A relatively fast kinetics for the striatal region was observed, followed by a subsequent moderately fast washout. The regional in vivo distribution of this radiotracer was in agreement with the known distribution of PDE10A, being found predominantly in the putamen followed by the caudate nucleus, ventral striatum and substantia nigra. Compared with the activity in the striatum, the cortical and cerebellar activity were more than 10-fold lower. Later, Ooms et al. have investigated the effect of alterations in cAMP levels on [^18^F]JNJ-42259152 binding to PDE10A in the striatum homogenates. Increased affinity of this radiotracer for PDE10A was observed in the presence of cAMP, which seems to have an important role in the allosteric regulation of PDE10A [62].

**Figure 14:**
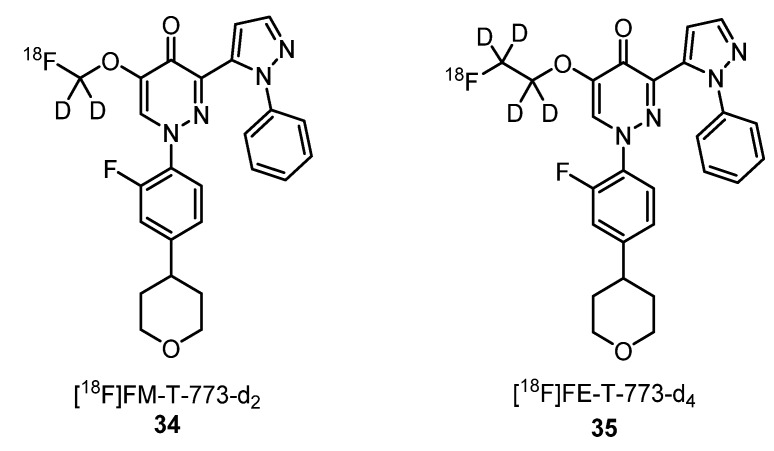
PET radiotracers **34** and **35** for in vivo phosphodiester-10A (PDE10A) evaluation in nonhuman primates’ brains [63].

Stepanov et al. described the synthesis of two [^18^F]-labeled PET radioligands to target PDE10A, the [^18^F]FM-T-773-d_2_ (**34**) and [^18^F]FE-T-773-d_4_ (**35**) (Figure 14), and their in vivo evaluation in nonhuman primates [63]. High brain uptake was measured for both radioligands and a fast washout. Specific binding reached the maximum after 30 min for [^18^F]FM-T-773-d_2_ (**34**) and after 45 min for [^18^F]FE-T-773-d_4_ (**35**). On account of brain uptake specific binding and kinetics, [^18^F]FM-T-773-d_2_ (**34**) was considered the more promising PET radioligand for further clinical evaluation.

#### 2.1.8. Translocator Protein Receptor Ligands

The translocator protein (TSPO) receptor is an 18 kDa protein organized around five large transmembrane α-helices and located on the mitochondrial outer membrane [2]. This protein has a key role in the regulation of several cellular processes: steroid biosynthesis, cholesterol metabolism, apoptosis and cellular metabolism [64]. TSPO is highly expressed in organs involved in steroid synthesis as adrenal glands, testis, ovaries and pituitary glands [65]. In the central nervous system (CNS) and liver, TSPO expression is modest. However, in the case of acute or neurodegenerative pathologies associated with microglia or astrocytes, levels of TSPO in the brain are upregulated. The upregulation of this protein is directly correlated with the degree of neuronal damage. For these reasons, TSPO is considered a very promising target for the early imaging of neuroinflammation [66] and a possible indirect marker of neuronal loss progression, multiple sclerosis and AD [66,67,68] and has high relevance in neuroscience. The expression of this protein is also elevated in several cancers: colon, breast, glioma, prostate, colorectal, liver and ovary cancer, relating TSPO with disease progression and survival [2,64,65,69]. These evidences increased the interest in TSPO and led to the development of several radiolabeled ligands for the evaluation of the expression of this protein and detection of some of the aforementioned diseases by PET imaging.

**Figure 15:**
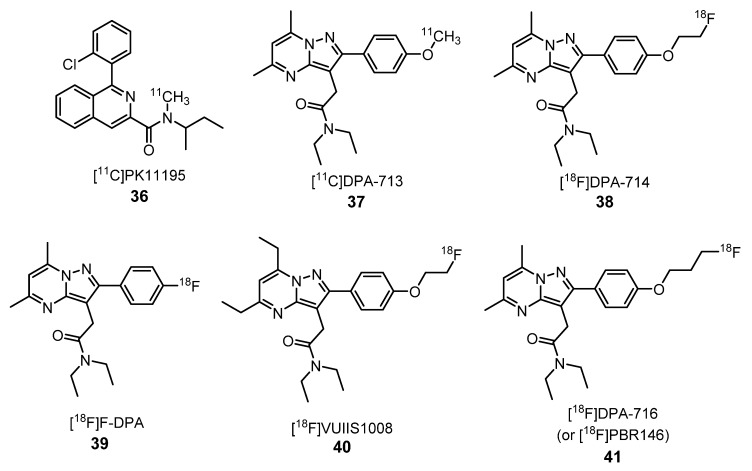
[^11^C]PK11195 (**36**) and pyrazolopyrimidine-derived radiotracers **37**–**41** for translocator protein (TSPO) PET imaging [72,73,74,75,76,77,78,79,80,81,82].

Since the discovery of the first nonbenzodiazepine ligand for TSPO, the isoquinoline carboxamide PK11195 (**36**) [70,71,72], several families of compounds were evaluated as TSPO ligands—among them, [^11^C]DPA-713 (**37**), [^18^F]DPA-714 (**38**), [^18^F]DPA (**39**), [^18^F]VUIIS1008 (**40**) and [^18^F]DPA-716 (or [^18^F]PBR146) (**41**), which are pyrazolo[1,5-*a*]pyrimidine acetamides (Figure 15) [72,73].

Chauveau et al. compared compound **38** with **36** and **37** using a rat model of acute neuroinflammation. (*R*,*S*)-α-amino-3-hydroxy-5-methyl-4-isoxazolopropionic acid (AMPA) was used to provoke neuroinflammation. Compounds **37** and **38** were specifically localized in the neuroinflammatory site with a similar signal-to-noise ratio in vitro. However, the fluorine-labeled tracer **38** achieved a higher bioavailability in the brain, higher uptake and higher binding potential than the other radiotracers. In the reference zone (contralateral area), a lower uptake of **37** and **38** were found when compared with **36**. These results showed that **37** and **38** have lower nonspecific bindings than **36** [74].

Compound **38** was also used to monitor the TSPO levels after the injection of some antibiotic like minocycline. This drug inhibits the activation of microglial cells [75]. Furthermore, the radiotracer **38** was used on studies of rodent models of excitotoxicity, herpes encephalitis [76], astrocytic activation, excitotoxically lesioned nonhuman primate brains, abdominal aortic aneurysm [77], rheumatoid arthritis [78,79] and neuroinflammatory changes in the brain after morphine exposure [65,67]. It demonstrated a good uptake in the primate brain and an eight-fold higher uptake in the lesioned striatum of a quinolinic acid-lesioned rat model of activated microglia. Both **37** and **38** showed better imaging properties than **36** in the striatum of lesioned rats. A study using a rat model of herpes simplex encephalitis (HSE) suggested that **38**, as an agonist of TSPO, is potentially suitable for visualizing mild neuroinflammation [76].

Kuszpit et al. demonstrated that [^18^F]DPA-714 (**38**) is a sensitive tool for the detection of neuroinflammation, induced by Zika virus (ZIKV) infection, using a mice model of ZIKV neurological disease. Moreover, the evaluation of therapeutics being developed for the treatment of the disease is also possible by [^18^F]DPA-714 (**38**) imaging [80].

In 2017, Keller et al. compared the activity of **38** with its analogue [^18^F]F-DPA (**39**) (Figure 15), which presents the radionuclide directly linked to the phenyl ring. This study showed that compound **39** is metabolically more stable than its analogue **38** in rats’ brains, being regarded as a promising TSPO radiotracer, because it shows a higher ratio between specific and nonspecific binding [81]. Later, in 2018, the same author described the potential of [^18^F]F-DPA **39** to visualize activated microglia at an early stage of AD pathology. The in vivo PET imaging and ex vivo brain AGR data indicated and increased uptake of this radiotracer **39** with age in the brains of transgenic animals (APP/PS1-21 mouse models of AD) in comparison with wild-type animals [68].

In 2012, Tang et al. used glioma-bearing rats to study the feasibility of using [^18^F]DPA-714 (**38**) for the visualization of TSPO expressing in brain tumors. This PET tracer showed to be highly specific to TSPO in glioma cell line homogenates. In vivo studies showed a higher uptake of **38** in tumor tissues than in other brain regions, suggesting that this compound could be used as a novel predictive cancer imaging tool [82]. In 2013, the same author carried out the synthesis and structure-activity relationship study of pyrazolopyrimidine-derived radioligands, presenting different substituents, namely at the 5, 6 and 7 positions, in order to find the more robust PET ligand. The best result was achieved with compound [^18^F]VUIIS1008 (**40**) (Figure 15), which showed a negligible binding in normal brains but a much higher binding in tumor tissues [64]. This result demonstrated that this radiotracer is a promising PET ligand for TSPO in tumors.

**Figure 16:**
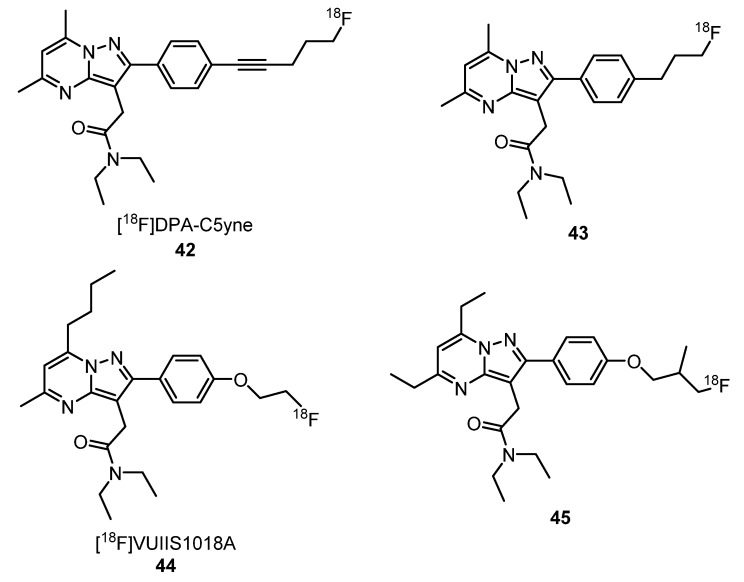
Pyrazolopyrimidine-derived radiotracers **42**–**45** for TSPO PET imaging [83,84,85,86,87].

In 2014, Médran-Navarrete et al. evaluated a new ^18^F-labeled analogue of [^18^F]DPA-714 (**38**), the [^18^F]DPA-C5yne (**42**) (Figure 16), as a TSPO radioligand. In vitro studies revealed that **42** was stable in plasma at 37 °C for at least 90 min. AGR studies, using slices of AMPA-lesioned rat brains, showed a high specificity of binding and selectivity for TSPO, highlighting the potential of **42** as a radiotracer for TSPO [83]. One year later, Damont et al. synthetized a series of novel pyrazolo[1,5-*a*]pyrimidines and evaluated, in vitro, their affinity, lipophilicity and metabolism. Based on the results obtained, two of the synthetized compounds were chosen for ^18^F-radiolabeling affording ligands [^18^F]DPA-C5yne (**42**) and (**43**) an analogue of [^18^F]DPA-714 (**38**), where the oxygen atom was replaced by a methylene group (Figure 16). Neuroinflammation PET images, using anesthetized Wistar rats seven days after AMPA-induced brain lesions in the right striatum, showed that both radiotracers **42** and **43** have a high in vivo-specific binding for TSPO. Compound **43** presented an ipsilateral-to-contralateral ratio of 3.57 ± 0.48 comparable to **38** (3.71 ± 0.39), and **42** showed an ipsilateral-to-contralateral ratio of 4.62 ± 0.44. These results suggest that both **42** and **43** are appropriate for neuroinflammation imaging [72].

Recently, Tang et al. synthetized a new TSPO PET tracer [^18^F]VUIIS1018A (**44**), an analogue of [^18^F]DPA-714 (**38**) where the 7-methyl group of the pyrazolopyrimidine ring was replaced by a *n*-butyl group (Figure 16), and evaluated its behavior in a preclinical model of neuroinflammation. These authors concluded that this structural modification increased the lipophilicity of **44** compared with **38** (3.7 vs. 2.4, respectively). After 60 min of injection of **44**, more than 85% of this probe was intact, indicating that it is very stable in the brain. It is noteworthy that, for **38**, just 50% of the probe was intact after 60 min of injection. In vivo blocking experiments and in vitro AGR assays confirmed a high binding specificity of **44** for TSPO, showing that it can be a promising TSPO PET tracer [84]. Tang et al. developed a preclinical evaluation to image glioma. In this work, **44** exhibited a lower accumulation in healthy brains, what was regarded as an advantage to distinguish lower-grade gliomas. Compared with [^18^F]DPA-714 (**38**) and [^18^F]VUIIS1008 (**40**), **44** had an improved tumor-to-background ratio, a higher specific-to-nonspecific binding ratio and a higher tumor-binding potential. These results showed that **44** is a promising candidate to detect tumors with modest TSPO expression [85].

A series of novel 2-phenylpyrazolo[1,5-*α*]pyrimidin-3-ylacetamides were synthesized, and their in vitro binding affinities for TSPO and lipophilicity (log P_7.5_) were evaluated using [^18^F]DPA-714 (**38**) as the control. Based on the results of these assays, the tosylated precursor was selected for radiofluorination, affording **45** (Figure 16). Using LPS induced neuroinflammation rat models, a dynamic micro-PET study was performed and demonstrated a higher uptake of **45** in the ipsilateral region and a higher ratio of target-to-background than **38**. These results suggest that **45** can be a promising PET probe candidate for TSPO imaging [86].

In 2018, Verweij et al. measured the cellular response in patients after an acute coronary syndrome by PET imaging using [^18^F]DPA-714 (**38**) as a probe. TSPO receptor is highly expressed in myeloid cells. Using a PET tracer with a high affinity for the TSPO receptor as **38** was possible to determine the hematopoietic activity. In the acute phase, the treatment with **38** revealed a higher uptake in the bone marrow and the spleen. Three months after, the bone marrow uptake decreased to levels comparable to the healthy control. On the other hand, the spleen uptake remained elevated [87].

## 3. Conclusions

Considering that ^18^F is the most suitable radionuclide for routine PET imaging and that pyrazoles are a key motif in medicinal chemistry and drug design, we made a compilation of [^18^F]pyrazole-derived imaging probes that have been developed and evaluated in the last 20 years. Regarding the examples presented herein, it is evident that pyrazoles are important scaffolds for the development of radiotracers for the diagnosis of several pathologies. In fact, pyrazoles interact with remarkable targets, namely with adenosine receptors, cannabinoid receptors, cyclooxygenase enzymes, dopamine receptors, glucocorticoid receptor, insulin-like growth factor-1 receptor, phosphodiester-10A enzyme and the translocator protein receptor. Among the probes presented herein, most are cannabinoid ligands and dopamine ligands, with a huge potential for brain imaging and TSPO ligands, namely pyrazolopyrimidine-derived radiotracers, that have shown important applications for the imaging of neuroinflammation and cancer.

The successful use of the described pyrazoles-imaging probes in clinics may help to increase the understanding of several diseases such as AD, PD, Huntington’s disease, atherosclerosis, neuropsychiatric disorders, neuroinflammation, cardiovascular diseases and cancer, among others, and to identify ways to improve therapy. In this sense, the pyrazoles described herein can be regarded as potential theragnostic agents and as important templates for the development of novel radiotracers with improved properties for PET imaging.
